# Platelet Mechanosensing of Collagen Matrices

**DOI:** 10.1371/journal.pone.0126624

**Published:** 2015-04-27

**Authors:** Matthew F. Kee, David R. Myers, Yumiko Sakurai, Wilbur A. Lam, Yongzhi Qiu

**Affiliations:** 1 Wallace H. Coulter Department of Biomedical Engineering, Georgia Institute of Technology and Emory University, Atlanta, Georgia, United States of America; 2 Department of Pediatrics, Division of Pediatric Hematology/Oncology, Aflac Cancer Center and Blood Disorders Service of Children’s Healthcare of Atlanta, Emory University School of Medicine, Atlanta, Georgia, United States of America; 3 Winship Cancer Institute of Emory University, Atlanta, Georgia, United States of America; 4 Parker H. Petit Institute of Bioengineering & Bioscience, Georgia Institute of Technology, Atlanta, Georgia, United States of America; University Hospital Medical Centre, GERMANY

## Abstract

During vascular injury, platelets adhere to exposed subendothelial proteins, such as collagen, on the blood vessel walls to trigger clot formation. Although the biochemical signalings of platelet-collagen interactions have been well characterized, little is known about the role microenvironmental biomechanical properties, such as vascular wall stiffness, may have on clot formation. To that end, we investigated how substrates of varying stiffness conjugated with the same concentration of Type I collagen affect platelet adhesion, spreading, and activation. Using collagen-conjugated polyacrylamide (PA) gels of different stiffnesses, we observed that platelets do in fact mechanotransduce the stiffness cues of collagen substrates, manifesting in increased platelet spreading on stiffer substrates. In addition, increasing substrate stiffness also increases phosphatidylserine exposure, a key aspect of platelet activation that initiates coagulation on the platelet surface. Mechanistically, these collagen substrate stiffness effects are mediated by extracellular calcium levels and actomyosin pathways driven by myosin light chain kinase but not Rho-associated protein kinase. Overall, our results improve our understanding of how the mechanics of different tissues and stroma affect clot formation, what role the increased vessel wall stiffness in atherosclerosis may directly have on thrombosis leading to heart attacks and strokes, and how age-related increased vessel wall stiffness affects hemostasis and thrombosis.

## Introduction

During a vascular injury, platelets are exposed to and adhere to subendothelial proteins such as collagen, initiating the process of clot formation. After initial adhesion, platelets then spread and activate, undergoing a myriad of changes including the activation of integrin α_IIb_β_3_ [1, 2]_,_ the release of granules to recruit other platelets [3, 4] and phosphatidylserine (PS) exposure to initiate the coagulation cascade [5, 6]. Biochemically and biologically, these processes are well characterized. However, much less is known regarding how the mechanical properties of the subendothelial matrix affect the platelet adhesion and activation.

In other cell types such as endothelial cells and fibroblasts, the microenvironmental mechanical cues, such as stiffness, of the underlying substrate alter the physiological processes of those cells via mechanotransduction [7–9]. Interestingly, a significant portion of the cytoskeletal machinery that mediates mechanobiology in nucleated mammalian cells is also present in platelets. As such, platelets, albeit anucleate and “simpler” than other cell types, may also mechanotransduce the signals of the underlying matrix. Indeed, we have recently observed that the stiffness of fibrin/fibrinogen substrates modulates platelet adhesion, spreading, and activation [10]. Here, we extend our work to investigate if and/or how platelets mechanosense collagen matrices and what affects this process may have on platelet physiology.

As recent studies have documented that the stiffness of subendothelial collagen correlates with the degree of different conditions, such as atherosclerosis or increasing age, these studies are clinically relevant [11–15]. To be able to understand how biomechanical factors influence clot formation in diseased states, a comprehensive understanding of the effect of increased subendothelial stiffness on platelet adhesion, spreading, and activation is therefore crucial.

To that end and to decouple the mechanical cues from biological and biochemical factors that may be involved, we covalently conjugated collagen on polyacrylamide (PA) gels of varied stiffnesses and investigated how platelets biologically respond to these differences in substrate stiffness. We observed that on collagen-conjugated PA gels with stiffness of over 5 kPa, the average spreading area of platelets adhered onto those gels was significantly increased as compared to those adhered on collagen-conjugated PA gels softer than 5 kPa. No difference was detected in the number of adhered platelets on PA gels of different stiffnesses. Interestingly, PS exposure, a marker of platelet activation and “procoagulabilty”, increased with increasing PA stiffness. To investigate the underlying mechanisms of these phenomena, we then conducted studies using pharmacologic cytoskeletal inhibitors, differential calcium levels and inhibitors of ADP signaling and thromboxane A2 generation.

## Material and Methods

### Fabrication of collagen-conjugated PA gels

12 mm glass slides were exposed to oxygen plasma for 1 minute and were silanized with 10% (3-aminopropyl)—trimethoxysilane (Sigma-Aldrich) in 95% ethanol for 45 minutes. Afterwards, the glass slides were treated with 0.5% glutaldehyde solution (Sigma-Aldrich) for 30 minutes. For gel fabrication, different ratios of acrylamide (Sigma-Aldrich) and bis-acrylamide (Sigma-Aldrich), crosslinked with 0.1% ammonium persulfate and tetramethylethrlenediamine (Sigma-Aldrich), were used to create different PA gels of varying stiffness according to standard protocols [16]. The PA gels were then placed into HEPES buffer solution (50 mM, pH = 8.25) combined with 0.25 mg/ml Sulfo-Sanpah (Pierce Biotechnology) and exposed to UV light for 10 minutes. Gels were then washed and incubated at 37°C with HEPES buffer along with 100 μg/ml rat tail collagen type I overnight, providing a constant surface layer of conjugated collagen on PA gels of varying stiffness [17].

### Isolation of washed human platelets

The protocol was approved by Emory University IRBs and written informed consent was received from all participants. To isolate platelets, 3 ml of blood was drawn into conical tubes with ACD anticoagulant and spun at 150 G’s for 15 minutes without a brake or acceleration. Platelet-rich plasma was then collected and centrifuged again at 900 G’s with 10% (v/v) ACD for 5 minutes. Platelet-poor plasma was then removed and the remaining platelet pellet was suspended in Tyrode’s buffer with 0.1% BSA. Platelets were then diluted to 5.5 x 10^5^ platelets/ml and 1 mM of Ca^2+^ and 2 mM of Mg^2+^ were added to the suspension of platelets, which were incubated onto the collagen-conjugated PA gels for 1 hour at 37°C. 50 minutes into the incubation, a fluorescent membrane dye, CellMask Orange (1:1000, Life Technologies), was added to the solution. The PA gels were then washed with PBS 3 times and fixed for 10 minutes with 4% PFA. For experiments using pharmacologic cytoskeletal inhibitors, ML-7 (30 μM, Sigma-Aldrich), Y-27632 (10 μM, Sigma-Aldrich), or latrunculin A (2 μM, Sigma-Aldrich) was used to pre-treat washed platelets for 1hour before incubation on collagen-conjugated PA gels. To study the effect of ADP and thromboxane A2 signaling on platelet mechanosensing on collagen, washed platelets were also pre-treated with apyrase (0.05 U/mL), aspirin (1 mM) or clopidogrel (250 μM) for 1hour before incubation on collagen-conjugated PA gels.

### Measuring platelet adhesion, spreading and activation on collagen matrices

Adherent platelets on PA gels were imaged with epi-fluorescence microscopy (Nikon TE2000-U) using a 20x objective. The number of adherent platelets in each image was counted using ImageJ, and the numbers from three images were averaged for each gel. Adherent platelets were also imaged with Z stack using confocal microscopy (Zeiss LSM700). Similarly, three representative images were taken for each gel. The Z-stack images were then flattened, and the spreading area of each platelet was also measured using ImageJ. Spreading area of platelets in all three images was averaged for each gel.

To visualize platelet activation as measured by integrin α_IIb_β_3_ activation, PAC-1-FITC (BD Science) was applied to adherent platelets for 5 minutes before fixation. Similarily, to visualize PS exposure on adhered platelets, Annexin V-Alexa Fluor 488 (5% v/v, Life Technologies) was incubated with adherent platelet for 5 minutes before fixation. Stained platelets were then imaged with Z stack using confocal microscopy. The same laser light intensity and gain settings were used for all images of either PAC-1-FITC or Annexin-V-Alexa Fluor 488 staining. The Z-stack images were flattened, and the average fluorescence intensity over the spreading area of each adherent platelet was measured with ImageJ.

### Statistics

The data in the figures are presented with ± standard deviation bars. Using Minitab Statistical Analysis Software these groups were analyzed for differences using Student’s t-test with a p-value of less than 0.05 denoting statistical significance.

## Results

### Spreading area of adhered platelets is mediated by stiffness

**Fig 1:**
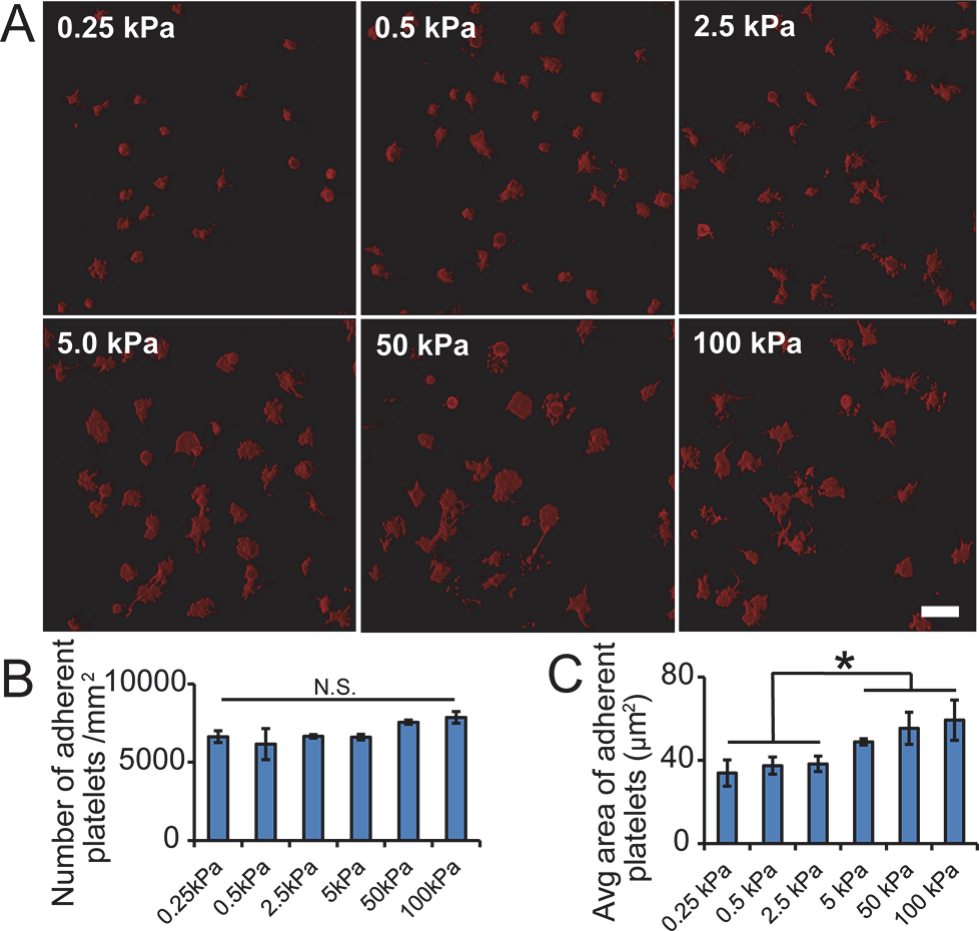
Substrate stiffness mediates platelet adhesion but not platelet spreading on collagen. A) Representative confocal microscopy images of adherent platelets on collagen-conjugated substrates with varying stiffness. B) The average number and C) the average spreading area of adherent platelets on collagen-conjugated substrates of varying stiffnesses. Scale bar = 20 um. p< 0.05; n = 3 experiments; error bars = standard deviation.

Platelets were found to adhere and spread on the substrates of all stiffnesses. There was no significant difference in the number of adhered platelets on gels of varying stiffnesses. Regardless of the substrate stiffness, ~ 5000 to 9000 platelets/mm^2^ adhered on the PA gels after 1 hour incubation (Fig 1A and 1B). However, adherent platelets spread to different degrees on collagen-conjugated PA gels of varying stiffnesses. There were significant differences between spreading area on gel stiffness ranging from 0.25 kPa to 2.5 kPa compared to gels stiffer than 5 kPa (Fig 1A and 1C). Adherent platelets on gels softer than 5 kPa have a spreading area of ~30–40 μm^2^, while those on gels stiffer than 5 kPa have a spreading area of ~50–60 μm^2^ (Fig 1C).

### Actomyosin activity controls stiffness mediated platelet spreading on collagen

**Fig 2:**
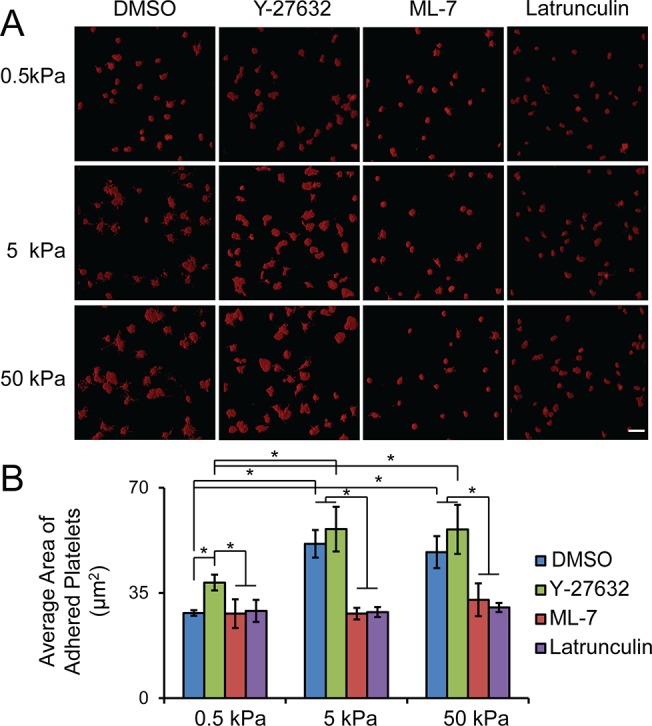
Substrate stiffness mediated platelet spreading on collagen is regulated by actomyosin activity. A) Representative confocal microscopy images and B) quantification of average spreading of adherent platelets on collagen-conjugated substrates of varying stiffnesses after pre-treatment with pharmacological inhibitors, ROCK, MLCK and latrunculin, respectively. Scale bar = 20 um; p< 0.05; n = 3 experiments; error bars = standard deviation.

Myosin is activated through two different pathways, involving MLCK and ROCK, respectively [18, 19]. Interestingly, inhibition of MLCK using ML-7, and inhibition of ROCK via Y-27632 resulted in differential effects on substrate stiffness-mediated platelet spreading on collagen-conjugated PA gels. Pre-treatment with ML-7 completely abolished the difference on platelet spreading, and adherent platelets showed minimal spreading on the substrates of all stiffnesses (Fig 2), while pre-treatment with Y-27632 showed no effect (Fig 2). These results indicate that stiffness-mediated platelet spreading on collagen is dependent on myosin activity through the MLCK signal pathway. More interestingly, for gel stiffnesses of 0.5 kPa though, Y-27632 treatment increased the platelet spreading area significantly (~40 μm^2^) compared to control conditions (~30 μm^2^) (Fig 2B). Pre-treatment with 2 μM latrunculin A, which disrupts actin polymerization, also decreased platelet spreading on both 5 and 50 kPa gels to a similar level as that on softer 0.5 kPa gels (Fig 2). This indicates that stiffness-mediated platelet spreading is also tightly regulated by actin polymerization. Taken together, these pharmacological inhibitor treatments indicate that stiffness-mediated platelet spreading on collagen is mediated by actomyosin activity.

### Ca^2+^ presence plays a critical role in the spreading of adhered platelets

**Fig 3:**
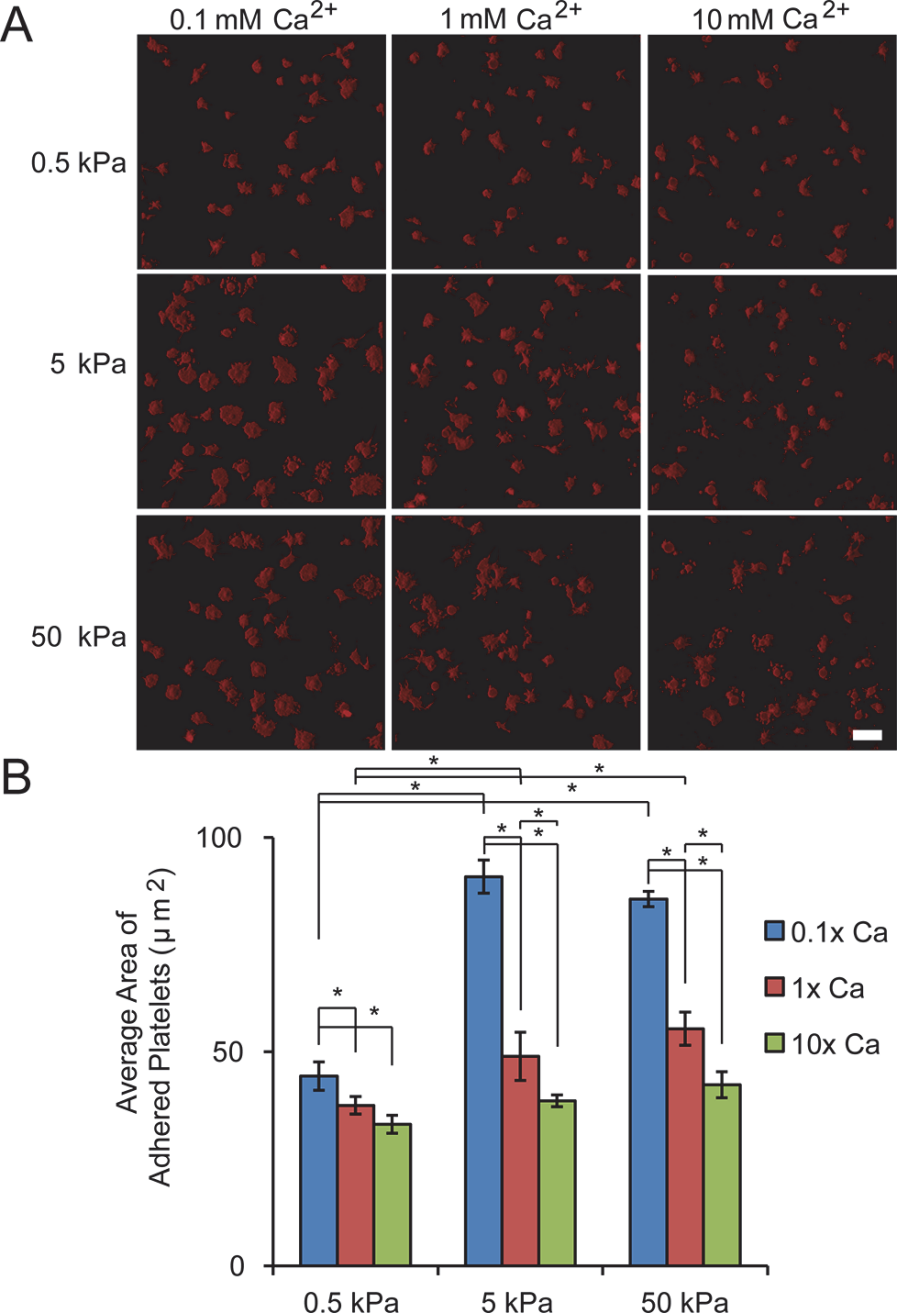
Extracellular Ca^2+^ concentration affects substrate stiffness mediated spreading on collagen. A) Representative confocal microscopy images and B) quantification of average spreading area of adherent platelets on collagen-conjugated substrates of varying stiffnesses when cultured with different Ca^**2+**^ concentrations. Scale bar = 20 um; p< 0.05; n = 3 experiments; error bars = standard deviation.

As activation via adhesion to collagen induces cytosolic calcium increase, calcium-dependent calpain activation, bleb formation and PS exposure [20–22], the influx of calcium could potentially compromise the stiffness mediated platelet spreading on collagen. To better understand how the presence of Ca^2+^ affects stiffness-mediated platelet spreading on collagen, platelets were incubated with different concentrations of calcium. Interestingly, a lower calcium concentration at 0.1 mM significantly increased platelet spreading on substrates of all stiffness compared to those at calcium concentration of 1 mM, while higher calcium concentrations (10 mM) decreased platelet spreading on substrates of all stiffnesses (Fig 3). Lower concentrations of calcium induced a more significant difference in platelet spreading between gels of 0.5 kPa and those stiffer than 0.5 kPa (Fig 3B). When 1 mM calcium was used, the platelet spreading on gels of both 5 and 50 kPa is about 1.6 times larger than that on gels of 0.5 kPa. However, this increased to ~ 2 fold when the calcium concentration was decreased to 0.1 mM, and decreased to ~1.2 fold as the calcium concentration was raised to 10 mM (Fig 3B). Taken together, the extracellular calcium concentrations directly affect the observed stiffness-mediated platelet spreading on collagen-conjugated PA gels.

### ADP and thromboxane A2 signaling antagonize substrate stiffness-mediated platelet spreading on collagen

**Fig 4:**
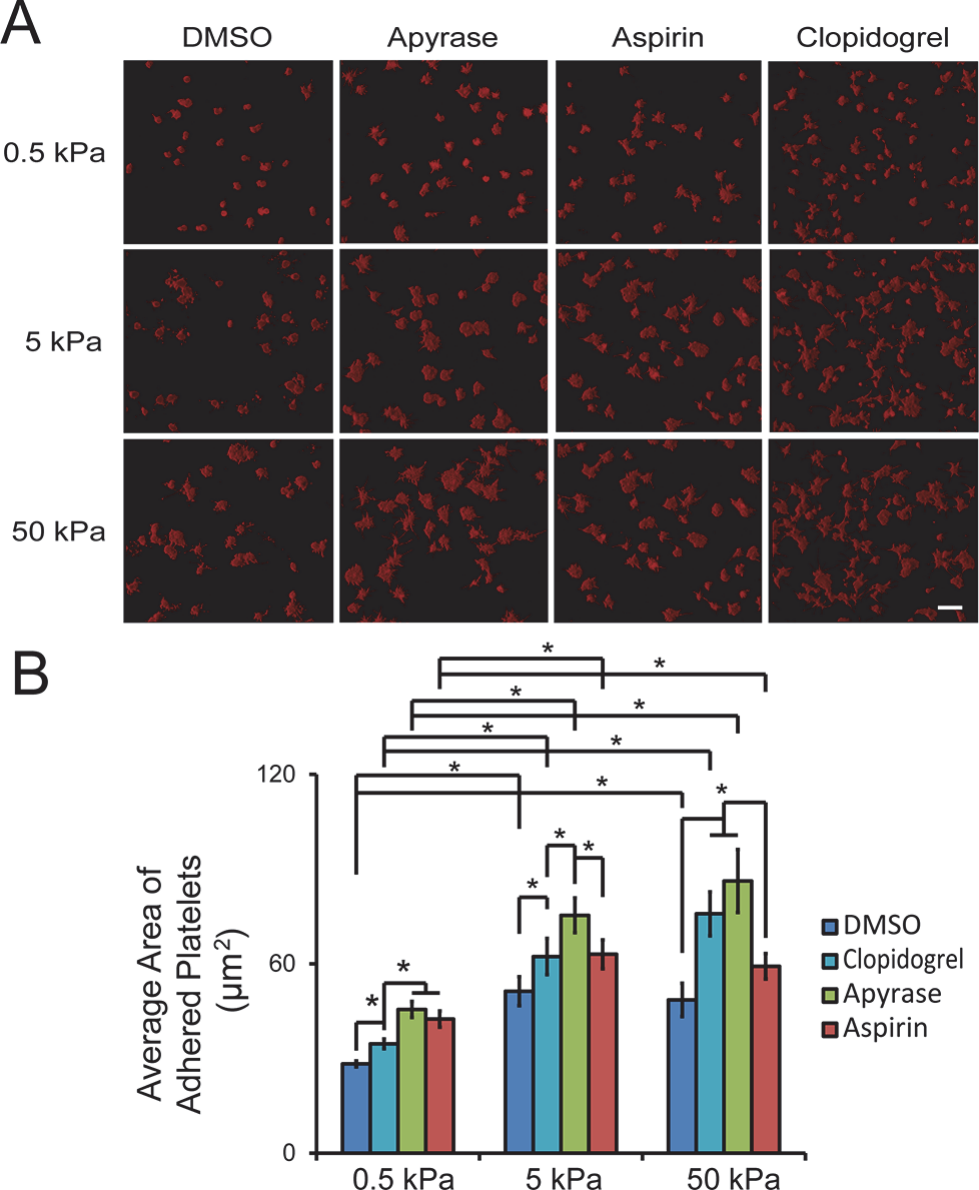
ADP and thromboxane A2 signaling antagonize substrate stiffness-mediated spreading on collagen. A) Representative confocal microscopy images and B) quantification of average spreading area of adherent platelets on collagen-conjugated substrates of varying stiffnesses after pre-treatment with apyrase, aspirin and clopidogrel. Scale bar = 20 um; p< 0.05; n = 3 experiments; error bars = standard deviation.

Adhesion of platelets on collagen can lead to ADP release and thromboxane A2 generation. To investigate the effect of ADP and thromboxane A2 on substrate stiffness-mediated platelet spreading on collagen, washed platelets were pretreated with apyrase, an ADP-degrading enzyme, and aspirin, a thromboxane A2 inhibitor. While apyrase and aspirin exposure increased platelet spreading on all the substrates regardless of the stiffness, the substrate stiffness- mediated platelet spreading was augmented as adherent platelets spread significantly more on both 5 and 50 kPa gels compared to that on 0.5 kPa gels (Fig 4A and 4B). More interestingly, apyrase treatment increased platelet spreading significantly more on stiffer substrates (both 5 and 50 kPa) compared to that with aspirin treatment. In addition, pretreatment of platelets with clopidogrel, an antagonist of ADP receptor P2Y_12_, shows a similar effect as apyrase (Fig 4A and 4B). Taken together, the results of inhibitor treatment suggested that ADP and thromboxane A2 signaling might antagonize the observed substrate stiffness-mediated platelet spreading (outside-in signaling through integrin α_2_β_1_) on collagen.

### Substrate stiffness mediates platelet activation

**Fig 5:**
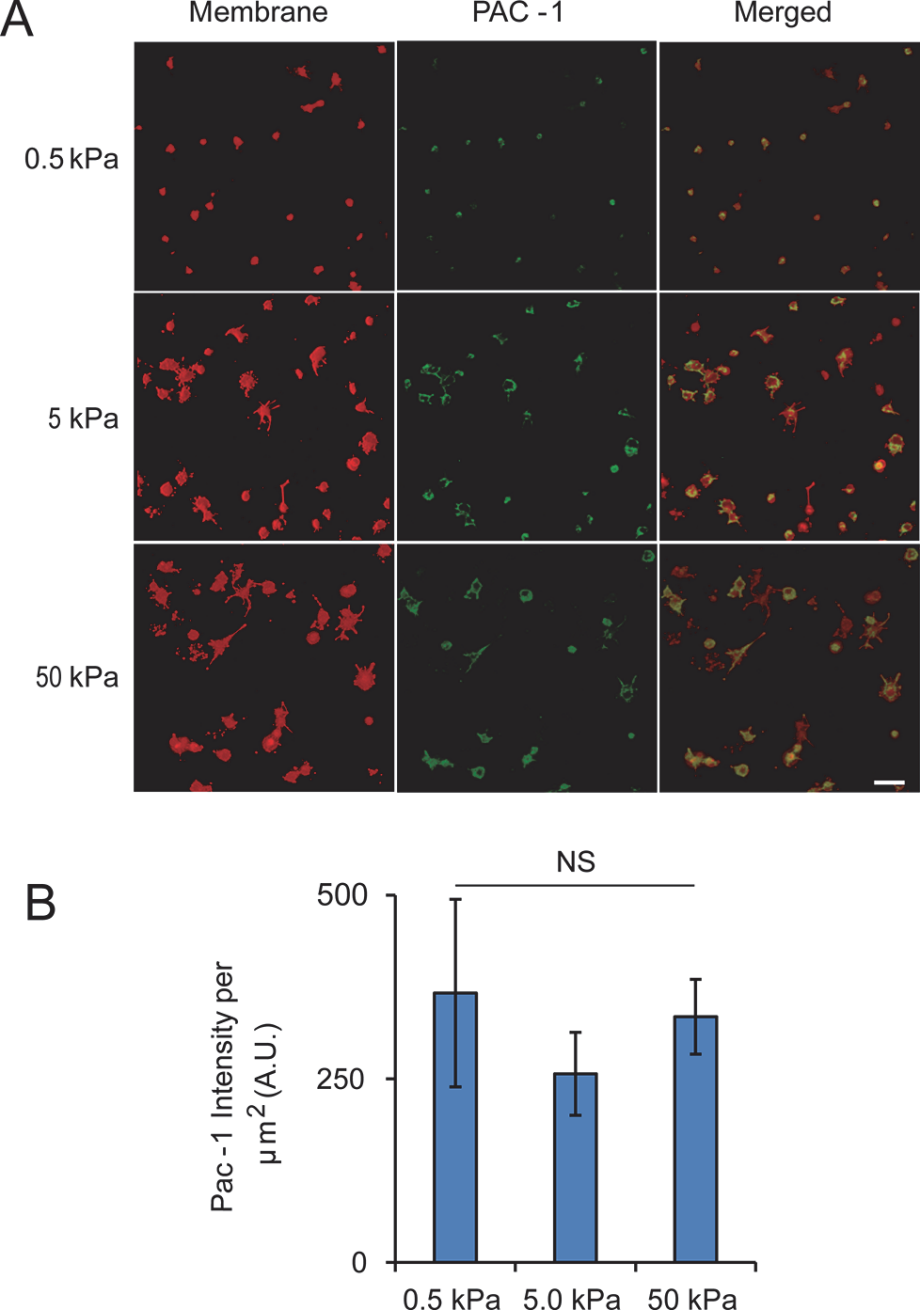
Integrin α_IIb_β_3_ activation of adherent platelets on collagen is not mediated by substrate stiffness. A) Representative confocal microscopy images of adherent platelets after staining with PAC-1-FITC and a fluorescent membrane dye (red). B) Average PAC-1-FITC intensity (a.u./μm2) showing no significant difference across all three stiffnesses. Scale bar = 20 um; p< 0.05, n = 3 experiments, error bar = standard deviation.

Binding to collagen can trigger outside-in signaling and activate α_IIb_β_3_ integrins for platelets aggregation and clot formation, and it can also leads to PS exposure and formation of procoagulant platelets [2, 3, 23, 24]. We therefore used activated α_IIb_β_3_ integrins and exposed PS as biomarkers to determine how substrate stiffness mediates platelet activation. Since the antibody, PAC-1-FITC, can specifically bind to activated α_IIb_β_3_ integrins, we quantified the fluorescence intensity to compare the platelet activation on substrate across all the stiffness (Fig 5A). Interestingly, the average intensity of platelets did not differ with the varying of the gel stiffness (Fig 5B).

**Fig 6:**
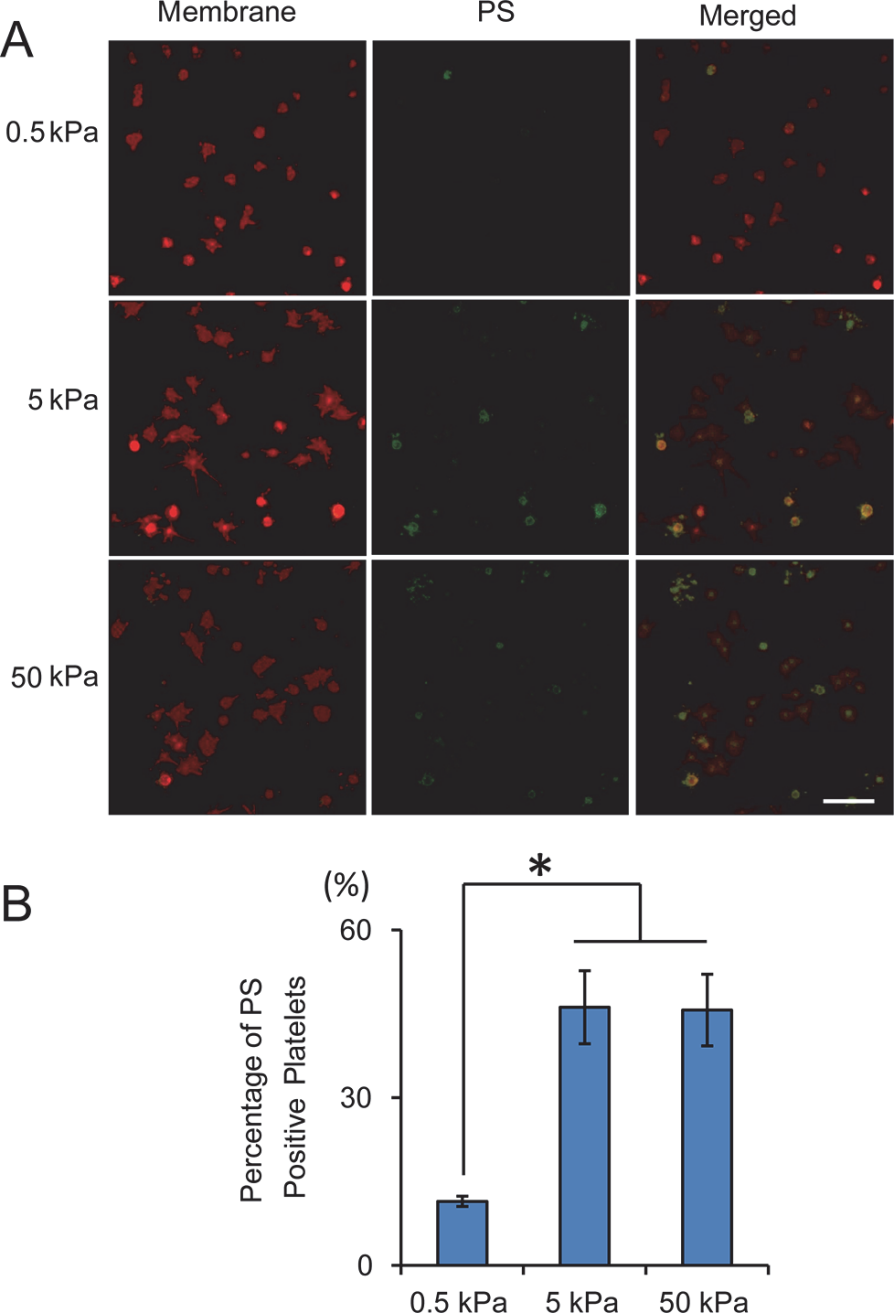
Substrate stiffness mediates PS exposure of adherent platelets on collagen. A) Representative confocal microscopy images of adherent platelets after staining with Annexin V-Alexa Fluor 488 and a fluorescent membrane dye (red). B) Stiffer substrates result in significantly higher percentage of PS-positive adherent platelets. Scale bar = 20 um; p< 0.05, n = 3 experiments, error bar = standard deviation.

We then tested the effect of substrate stiffness on procoagulant activity (PS exposure) of adherent platelets. Strikingly, the PS exposure of adherent platelets was mediated by the substrate stiffness (Fig 6A). On 0.5 kPa gels, 10% of the adhered platelets was stained PS positive. However, on 5 and 50 kPa gels the percentage of PS positive platelets increases to ~ 40–50% (Fig 6B).

## Discussion

Overall, our data show that platelets mechanosense the mechanical properties of the subendothelial collagen matrix of the vascular wall and exhibit differential degrees of spreading and activation on substrates of varying stiffness. To the best of our knowledge, this is first reported observation that platelets can mechanosense the underlying substrate stiffness when adhered to collagen. We used PA gels conjugated with the same density of rat type I collagen but varied in stiffness, ranging from 0.25 to 100 kPa, enabled decoupling of the biochemical from the mechanical cues of the subendothelium [8, 25, 26]. We found that platelet spreading was markedly decreased on PA gels of 5.0 kPa stiffness compared to that of stiffer gels. Interestingly, PS exposure was also significantly more increased in platelets adhered and spread onto stiffer (5.0 kPa and above) collagen-conjugated PA gels as compared to those on softer PA gels. Taken together, these data reveal that platelet mechanotransduction of the collagen microenvironment not only occurs, but also directly mediates downstream effects of platelet activation. PS exposure is a key component of platelet activation and is associated with procoagulant activity in which initiation of the coagulation cascade occurs on the platelet surface. Therefore, our results directly link mechanotransduction of collagen substrates with platelet physiology and clot formation.

Platelets bind to collagen through two transmembrane receptors, glycoprotein VI (GPVI) and integrin α2β1[24, 27–29], both of which play important roles in platelet adhesion and activation [30, 31]. GPVI is found to initiate the platelet adhesion and activate both integrin α2β1 and αIIbβ3 [32], which leads to stable platelet adhesion [33, 34]. Integrin α2β1 can also mediate outside-in regulation of platelet spreading on collagen [35]. Our observation that intensity of PAC1-FITC staining/integrin αIIbβ3 activity shows no differences on adherent platelets on collagen of varying stiffnesses, indicating that integrin αIIbβ3 activation is likely regulated by GPVI-collagen interaction and this interaction is not mediated by stiffness. As such, the effect of stiffness-mediated platelet spreading is likely regulated by integrin α2β1 through outside-in signaling.

Mechanistically, we observed that the substrate stiffness-mediated effects of platelet mechanotransduction of collagen-conjugated PA gels are driven by actin polymerization and MLCK. ROCK, however, is not involved in this process, suggesting that platelet mechanotransduction on collagen substrates involves a specific actomyosin pathway.

Interestingly, we also observed that extracellular Ca^2+^ also plays a role in this process: the lower the extracellular Ca^2+^ concentration, the more pronounced substrate stiffness-mediated platelet spreading is. This augmentation of the substrate-mediated effects at low Ca^2+^ levels may be due to the fact that the contractile actomyosin pathways are Ca^2+^-dependent [19]. Decreased Ca^2+^ levels may inhibit contractile pathways, which in turn may “un-inhibit” platelet spreading. We also observed that with higher concentration of Ca^2+^, more bleb formation appeared on adherent platelets on collagen. Adhesion to collagen has been reported to cause Ca^2+^ release and influx, and result in sustained high cytosolic Ca^2+^ level [20]. It also activates the Ca^2+^-dependent protease, calpain [20, 24], which cleaves cytoskeleton proteins and regulates bleb formation and shedding of microvesicles from activated platelets [21, 22]. It has recently been shown that platelets from calpain1^-/-^ mice exhibit enhanced spreading on both collagen and fibrinogen [36]. It is therefore likely that higher Ca^2+^ levels can cause faster or more calpain activity, which results in more bleb formation and reduces the stiffness-mediated effect on platelet spreading.

Adhesion of platelets on collagen can lead to ADP release and thromboxane A2 generation [37], which will amplify subsequent platelet activation. To further decouple these biochemical cues from outside-in platelet mechanosensing, we treated washed platelets with apyrase, aspirin or clopidogrel. Interestingly, inhibition of these signaling pathways augments substrate stiffness-mediated platelet spreading. Previous studies indicate that ADP and thromboxane A2 can elicit Ca^2+^ signaling of platelets adherent on collagen [38–40], which promotes bleb formation [20] and could potentially simultaneously reduce platelet spreading. Therefore, our data suggest that inhibition of ADP and thromboxane A2 may block the increase of cytosolic Ca ^2+^, thus decreasing bleb formation and increasing substrate stiffness-mediated platelet spreading, which is consistent with a previous report [20].

**Fig 7:**
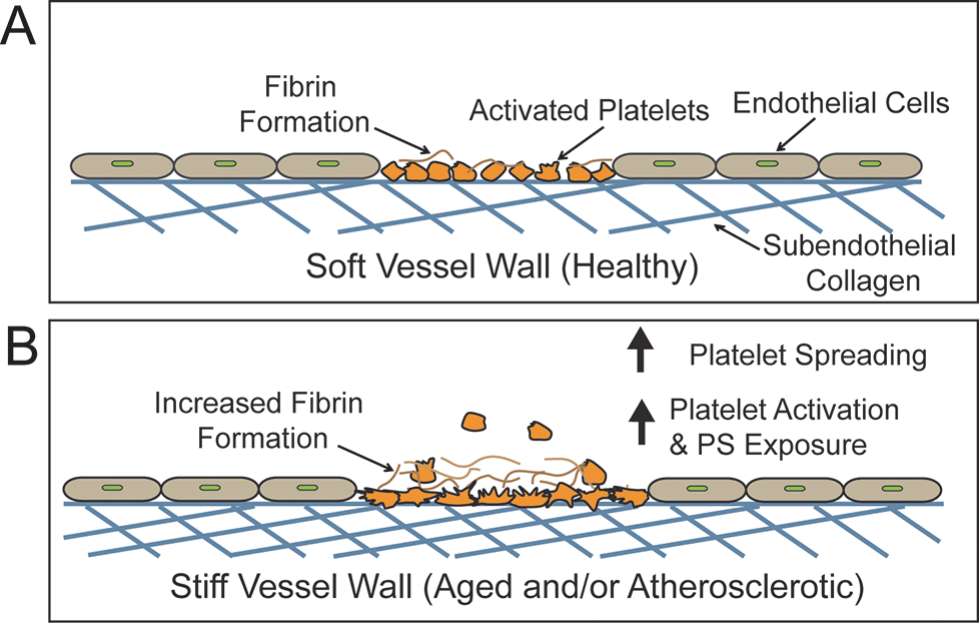
Platelet spreading and activation on exposed collagen at sites of vascular injury is mediated by the stiffness of underlying subendothelial collagen matrix. A) The vessel wall in healthy conditions is relatively soft, leading to less platelet spreading, platelet activation and PS exposure. B) The vessel wall in the elderly or atherosclerotic patients is comparatively stiffer, which will likely result in more platelet spreading, activation and PS exposure. The elevated PS exposure will then, in turn, cause increased fibrin formation, and recruit more platelets to the thrombus.

Overall, our study demonstrates that platelets have the capability to sense the mechanical properties of underlying collagen substrates and mechanotransduce these cues to alter their physiology to directly affect clot formation as in the case of PS exposure (Fig 7). These collagen substrate stiffness-mediated affects are driven by MLCK and actin polymerization as well as extracellular Ca^2+^ concentrations. These experiments have direct clinical relevance as vessel wall stiffness is noted to increase with age and with atherosclerosis [11–15]. Our data suggest that subendothelial collagen stiffness, in and of itself, would increase platelet spreading and activation (Fig 7B), and increase the risk of life threatening thrombosis. Our results also shed light on how different tissues, which all contain collagen, may harbor different levels of thrombogenicity simply based on their mechanical properties. In a broader sense, our data presented here, in addition to our previously published work, introduces how mechanics of the underlying substrate serve as another category of effectors that modulate platelet physiology, in addition to the biological and biochemical agonists that have been well characterized. In this work, we discovered that the mechanotransduction pathways in platelets are slightly different on collagen substrates as compared to fibrin/fibrinogen substrates. Ongoing studies will further our understanding of the role of platelet mechanotransduction of underlying substrates in hemostasis and thrombosis and how leveraging those pathways may lead to new therapeutic targets.
